# A multi-class fundus disease classification framework based on self-supervised contrastive learning and dual-branch network

**DOI:** 10.3389/fmed.2026.1932626

**Published:** 2026-10-09

**Authors:** Lili Cai, Keke Hou, Zhong Mo, Shining Ding

**Affiliations:** 1 School of Medicine and Health, Guangzhou Xinhua University, Guangzhou, China; 2 School of Information Engineering, Xinyang Agriculture and Forestry University, Xinyang, China

**Keywords:** contrastive learning, deep learning, dual-branch architecture, feature fusion, fundus disease classification, self-supervised learning

## Abstract

**Introduction:**

Fundus diseases including diabetic retinopathy, glaucoma, and age-related macular degeneration are primary causes of blindness worldwide. Manual interpretation of retinal images is time-consuming and heavily reliant on clinical expertise. Although deep learning has shown promise in automated fundus image analysis, its performance remains highly data-dependent, requiring large amounts of expert annotations. Moreover, single-architecture models may have limited capacity to capture the diverse pathological features present in fundus images.

**Methods:**

This study proposes a unified framework integrating self-supervised contrastive pre-training with a hybrid dual-branch architecture. Pre-training on unlabeled fundus images enables the model to learn generalizable representations, thereby reducing reliance on manual annotations. The dual-branch design combines EfficientNet-B4 for fine-grained detail extraction and MambaOut for large receptive field modeling. Their outputs are adaptively fused via a learnable cross-fusion module. A dendritic classifier is adopted to further improve classification performance. Comprehensive evaluations were conducted on two public datasets, OIHC and FIVES.

**Results:**

On the OIHC dataset, the proposed model achieved an F1-score of 96.32 ± 0.38% and a Kappa coefficient of 95.89 ± 0.43%. On the FIVES dataset, it attained 95.10 ± 0.54% and 93.49 ± 0.71%. The macro-average AUC exceeded 98% on both datasets. Ablation studies demonstrated the contribution of each component to the overall performance.

**Conclusion:**

This study indicates that combining self-supervised pre-training with a hybrid dual-branch architecture is a feasible approach for multi-class fundus disease classification. Interpretability analyses suggest that the model's attention regions partially overlap with clinically relevant pathological features. The framework achieved competitive performance on both datasets, showing its potential utility in scenarios with limited annotated data. Future work will focus on multi-center validation and model compression for lightweight architecture design.

## 1 Introduction

Vision is the primary channel through which humans perceive the external environment. Research indicates that approximately 70%–80% of external information is acquired through the visual system (1). Eye diseases represent a major global health concern. As reported by the World Health Organization, over 2.2 billion people worldwide are affected by blindness or low vision (2). Fundus diseases such as age-related macular degeneration (AMD), diabetic retinopathy (DR), and glaucoma represent a significant proportion of global blindness. These conditions progress through distinct stages, with their impact on vision varying at each stage. Early-stage fundus diseases often present with subtle symptoms. Without timely intervention, these conditions can result in irreversible damage. Early and accurate detection is essential, as timely treatment can help preserve vision and maintain quality of life (3).

Color fundus photography (CFP) is one of the most widely used clinical ophthalmic examination techniques. As a non-invasive imaging approach, it delivers high-definition views of the fundus structures (4). This modality has become a routine part of ophthalmic examinations. However, accurate diagnosis from color fundus images requires specialized physicians to perform detailed visual assessment. This process suffers from both substantial time costs and high susceptibility to subjective bias. Furthermore, underdeveloped and remote regions lack sufficient specialist expertise, which hampers timely and accurate diagnosis. These limitations make large-scale screening impractical in resource-constrained settings. Recent advances in deep learning have positioned this technique as a promising approach for automated fundus image assessment. In particular, convolutional neural networks (CNNs) have proven effective in classifying fundus diseases, achieving strong performance across DR (5), glaucoma (6), and AMD (7). Benbakreti et al. (8) applied pre-trained CNNs to four-class fundus classification and achieved 92.7% accuracy. Thanki (9) combined deep neural networks with traditional machine learning classifiers, showing the potential of hybrid strategies. These advances have established the feasibility of computer-aided diagnosis, but they also reveal persistent challenges. The first limitation concerns heavy dependence on annotated data. Training high-performance models requires large, expertly annotated datasets, yet medical annotation is costly, time-consuming, and relies on scarce expert knowledge (10). This severely limits model generalizability, particularly for rare diseases or underrepresented populations. The second limitation is architectural. A common practice in existing fundus disease classification is to adopt a single backbone, typically a CNN or a Transformer. CNNs excel at extracting local textures and fine-grained lesions, but they struggle with long-range spatial dependencies due to their limited receptive fields (11). Transformers capture global context effectively through self-attention, but their computational cost grows quadratically with image resolution, and they usually require large datasets to generalize well (12). These limitations suggest that a single architecture may be insufficient to capture the full spectrum of pathological features in fundus images. Recently, MambaOut has been proposed as an efficient alternative that removes the SSM component from Mamba and relies on large-kernel depthwise convolutions to enlarge the effective receptive field (13). The design exhibits strong performance and is well suited for the global feature extraction branch in a dual-branch architecture. This motivates us to explore a hybrid design that combines MambaOut with a complementary backbone for comprehensive feature extraction. To overcome these challenges, we propose a multi-class fundus disease classification framework that integrates self-supervised learning (SSL) with a hybrid dual-branch network. The framework operates in two stages. In the first stage, we employ SimCLR-based contrastive learning (14) on unlabeled fundus images to learn generalizable visual representations without manual annotations. This pre-training step helps reduce dependency on costly expert labels. In the second stage, we construct a dual-branch feature extraction architecture combining EfficientNet-B4 (15) and MambaOut as parallel backbones. This hybrid design provides richer feature representations for the subsequent classification task. Finally, we replace the standard linear classifier with a dendritic classifier (16) to enhance inter-class separability and improve classification performance on complex fundus disease categories. The main contributions of this work are summarized as follows:

(1) We propose a unified framework that integrates self-supervised contrastive pre-training with a dual-branch feature extraction network for multi-class fundus disease classification. Experimental results on the OIHC and FIVES datasets indicate that this design helps reduce the reliance on annotated data and maintains consistent performance across both datasets.
(2) This framework employs a dual-branch architecture combining EfficientNet-B4 with MambaOut. EfficientNet-B4 captures fine-grained local textures through depthwise separable convolutions and compound scaling, while MambaOut leverages large-kernel depthwise convolutions to aggregate information over broader spatial regions. This combination enlarges the receptive field while maintaining moderate computational efficiency for the classification task.
(3) We further introduce a feature-dimensional learnable cross fusion (FDLC) module. It adaptively integrates features from the two branches using a dimension-adaptive SE mechanism and a dynamic gated fusion strategy.
(4) In addition, a dendritic classifier is adopted for fundus disease classification. Inspired by the non-linear computation of biological neurons, this classifier aims to enhance discriminative power through non-linear decision boundaries. We evaluate the framework through ablation studies, comparative experiments, robustness tests, and interpretability analysis. These experimental results provide a preliminary basis for assessing the multi-branch framework in fundus disease classification tasks.

The organization of this paper is as follows. Section 2 reviews relevant research in fundus disease classification, self-supervised learning, and dual-branch architectures. Section 3 describes the proposed method and datasets. Section 4 provides the experimental results along with a detailed analysis. Section 5 discusses the findings and limitations. Section 6 concludes the paper and presents future work.

## 2 Related work

### 2.1 Deep learning-based fundus disease classification

Deep learning has become a key tool in automated fundus image analysis. Initial efforts focused primarily on single-disease detection. As a major cause of vision loss in the working-age population, DR is characterized by progressive microvascular damage. Gulshan et al. (5) demonstrated that a deep CNN could detect referable DR with sensitivity comparable to that of ophthalmologists. Naveen et al. (17) developed EffNet-SVM, combining EfficientNet features with SVM classification. This method achieved competitive performance on DR severity grading. MSAmix-Net (18) integrated multiscale convolution with self-attention, reporting 82.3% accuracy on five-class DR classification. The clinical presentation of glaucoma includes progressive optic neuropathy and associated visual field defects. Unlike DR, glaucoma diagnosis relies predominantly on optic disc evaluation, particularly the cup-to-disc ratio. Liao et al. (6) designed a model that generates glaucoma activation maps to enhance interpretability. Velpula and Sharma (19) introduced a multi-stage framework using five pre-trained CNNs with voting-based fusion. Nevertheless, early glaucomatous changes remain difficult to detect, as distinguishing normal variation from pathological cupping is a challenge even for deep learning models. AMD is a disease of the macula that primarily affects elderly individuals. It progresses from early drusen deposits to advanced stages, including geographic atrophy and neovascular AMD. Abd El-Khalek et al. (20) proposed a comprehensive CAD system for AMD diagnosis. They extracted appearance features at both local and global scales and applied weighted majority voting to an ensemble of classifiers. Their system categorized AMD into four stages and achieved an overall accuracy of 96.85%. In recent years, several studies have further advanced the development of fundus disease classification. Garcia-Atutxa et al. (21) benchmarked six DeepLabv3 configurations on the FIVES dataset using five independent random seeds to generate stratified splits, with the best model achieving a mean AUC of 0.979. Their work underscores that the difficulty of recognizing different fundus diseases varies considerably. Pachade et al. (22) introduced the RFMiD challenge, which provided a large-scale standardized evaluation framework for multi-disease detection. Duan and Tu (23) classified 16 fundus diseases using ultra-widefield images with a hybrid model combining DenseNet121 and XGBoost. They validated their method on an independent external test set. Their findings emphasized that performance differences between internal and external validation can be substantial. This highlights a key consideration for evaluating model generalizability. Despite their success, supervised deep learning methods rely heavily on large-scale labeled datasets. Given the high cost and specialized clinical expertise required for annotating fundus images, this annotation bottleneck remains a critical constraint for model development.

### 2.2 Learning with limited labels in medical images

To alleviate the annotation bottleneck, SSL has gained substantial attention in medical image analysis. SSL learns generalizable representations from unlabeled data through pretext tasks (10). As a representative SSL framework, SimCLR (14) introduced a contrastive framework that maximizes agreement between augmented views of the same image. SimCLR achieved competitive results on natural images, prompting researchers to adapt it to medical imaging. Azizi et al. (24) employed contrastive learning for dermatology classification with limited labeled data and showed clear improvements compared to supervised baselines. In fundus image classification, Liu et al. (25) proposed a general framework based on feature distillation from unlabeled fundus images for detecting multiple disorders. Semi-supervised learning also offers an alternative by leveraging both labeled and unlabeled data. Smitha and Jidesh (26) proposed a semi-supervised GAN for multiple retinal conditions, achieving 87% accuracy with limited annotations. Zhang et al. (27) introduced a multi-point attention SSL method for DR and achieved 92.6% accuracy. Malik et al. (28) proposed a dual-branch semi-supervised learning framework. With only 26% labeled data, it achieved an accuracy of 93.14% on four-class fundus classification and outperformed fully supervised baselines. Collectively, these findings highlight the potential of annotation-efficient learning strategies in clinical practice. However, existing SSL methods for fundus classification predominantly rely on single-backbone architectures and simple fusion strategies. More sophisticated feature extraction and integration mechanisms could further enhance performance.

### 2.3 Dual-branch architectures in medical image analysis

Hybrid architectures combining multiple feature extraction pathways offer a useful strategy in medical image processing. These models integrate complementary representations from different backbones, often outperforming single-architecture designs. Hybrid CNN-Transformer models combine the local texture extraction strength of CNNs with the long-range dependency modeling ability of Transformers. Such models have been widely used in medical image analysis (29). He et al. (30) proposed vision KAN-Transformer, combining ConvKan and local-global attention branches for medical image classification. The double-branch convolutional transformer (31) employs an attention mechanism to capture fine-grained features while remaining effective on small medical datasets. Liu et al. (32) proposed a parallel dual-branch network called CNN-Trans for fundus classification. The CNN-LSTM branch extracts local features with coordinate attention, and the ViT branch captures global dependencies through self-attention. Chen et al. (33) developed ViResGF-Net, a model that integrates ResNet50 and ViT branches with a gated fusion unit. It achieved an accuracy of 93.56% on multi-label fundus classification. Malik et al. (28) introduced a dual-branch framework for retinal disease classification. While these works have demonstrated the potential of dual-branch architectures, most still rely on simple concatenation or fixed-weight fusion strategies, which may not adapt to the varying importance of different feature sources across samples. Adaptive fusion mechanisms that dynamically weight branch contributions remain relatively underexplored in fundus image analysis.

Based on this review, there are two key research gaps. First, existing dual-branch architectures predominantly rely on non-adaptive fusion strategies that cannot adjust to sample-specific variations. Second, the integration of self-supervised pre-training with dual-branch architectures remains largely unexplored in the fundus domain. To address these gaps, we propose a unified framework combining SimCLR pre-training with a dual-branch architecture featuring EfficientNet-B4 and MambaOut. Our FDLC fusion module adaptively weights branch contributions, and the dendritic classifier further enhances discriminative power.

## 3 Materials and methods

### 3.1 Overview

Figure 1 illustrates the framework of our proposed system. The system uses a dual-branch feature extraction module combining EfficientNet-B4 with MambaOut. We pre-train the system using contrastive learning to facilitate the recognition of various fundus diseases. The framework consists of two stages. Stage 1 focuses on model pre-training. We employ self-supervised contrastive pre-training on many unlabeled fundus images. This stage is beneficial for learning general visual representations. Stage 2 involves training and fine-tuning based on the parameters obtained from Stage 1. We train the model using end-to-end supervised learning on limited labeled data to adapt it to multi-class fundus disease classification. The model adopts a dual-branch architecture with two parallel pathways, and their features are integrated through the proposed FDLC module. This design enables the model to capture both local textures and global context. EfficientNet-B4 extracts fine-grained local textures and subtle lesion structures in high-resolution fundus images using depthwise separable convolutions and compound scaling (15). MambaOut removes the SSM component from Mamba and relies on large-kernel depthwise convolutions to enlarge the receptive field. This design enables it to achieve competitive performance on image classification (13). The hybrid architecture combines the strengths of EfficientNet-B4 and MambaOut for comprehensive feature representation.

**Figure 1:**
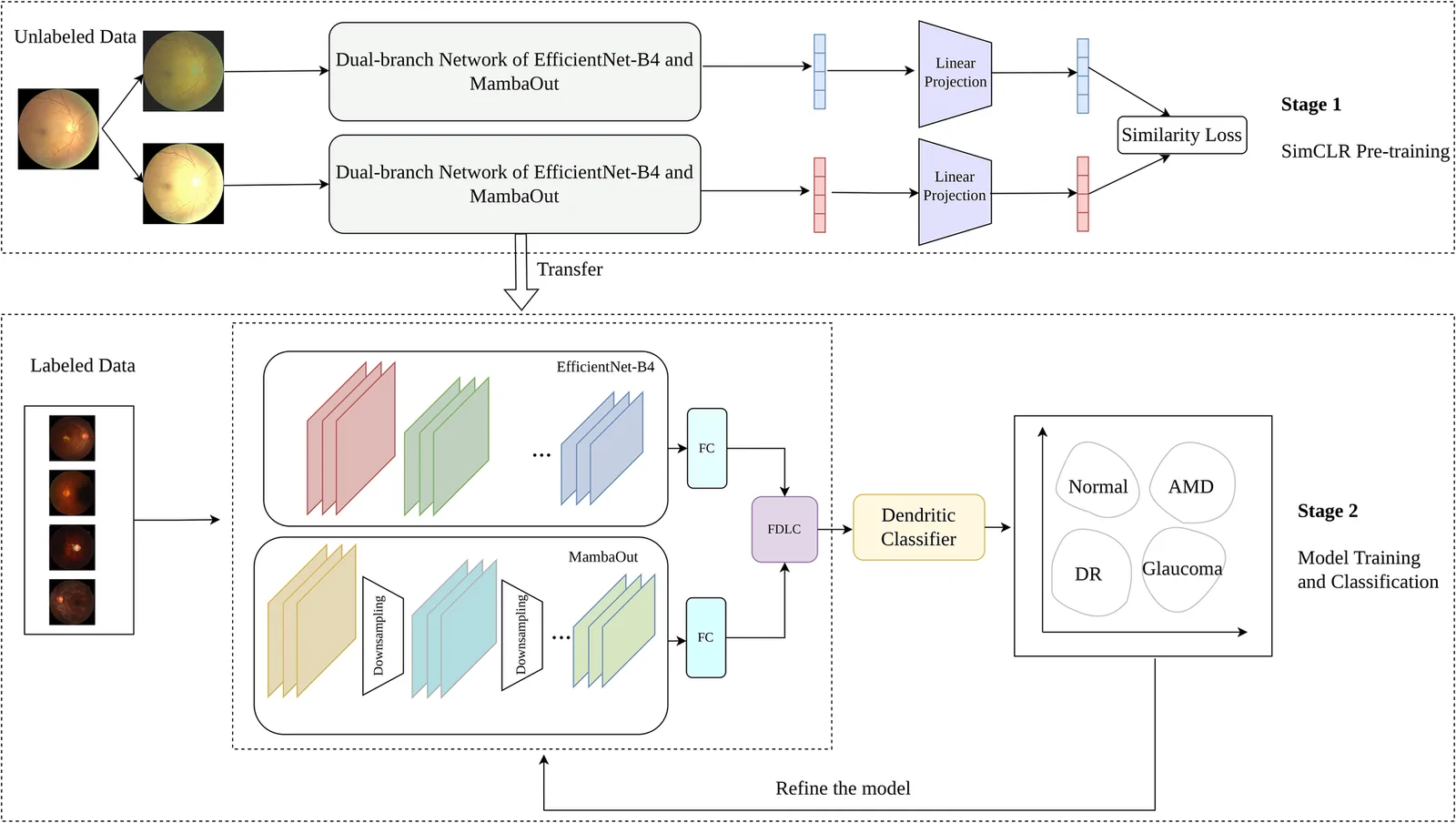
The framework of multi-class fundus disease classification.

Due to high heterogeneity and inter-class similarity in fundus disease features, traditional linear classifiers often struggle to separate complex decision boundaries clearly. To address this, the study draws inspiration from the dendritic classifier design proposed in DMixNet (16). This classifier is derived from dendritic neuron theory to enhance nonlinear processing capability. The adaptation for multi-class fundus disease aims to improve discriminative power of subtle pathological differences.

### 3.2 Datasets and image preprocessing strategy

The datasets used in this study include FIVES (34), OIHC (Ocular Imaging Health Cleaned), and ODIR-5K. The FIVES dataset was introduced in 2022 and contains 800 color fundus photographs with a resolution of 2,048 × 2,048 pixels. The images include DR, AMD, glaucoma, and normal fundus, with 200 images per category. The OIHC dataset was reconstructed based on the original OIH dataset. The OIH dataset integrates fundus images from multiple public sources. Since the specific composition and source documentation of this dataset were not fully available, we performed a systematic screening and cleaning process prior to use. The cleaning procedure consisted of three steps. First, we performed perceptual hash-based deduplication to identify identical or highly similar image copies between the original OIH dataset and the pretraining dataset ODIR-5K. Second, for visually similar images without exact hash matches, we performed an initial screening to flag potential overlap across datasets. Third, all suspected samples were independently reviewed by two researchers to confirm their source. Through this process, we removed images that overlapped with ODIR-5K at the image level. In addition, we excluded the cataract category from the dataset. Cataract is a lens condition rather than a retinal disease. In color fundus photographs, it primarily manifests as global blur, reduced contrast, and non-uniform illumination, which are visually similar to image quality degradation caused by variations in imaging devices, lighting conditions, or pupil size. This similarity poses a risk that a model may learn acquisition artifacts rather than clinically meaningful pathological features. Therefore, we removed all cataract samples to ensure the focus of this study on retinal diseases. To maintain dataset size and class balance, we supplemented the dataset with AMD and Normal samples. As summarized in Table 1, the AMD class contains 505 images from three sources: 155 from RFMiD 1.0 (35), 9 from RFMiD 2.0 (36), and 341 from OIA-ODIR (37). It should be noted that the official OIA-ODIR annotation reports 238 AMD cases at the patient level, whereas our count of 341 is at the image level. AMD samples were restricted to AMD-positive images, excluding those with comorbid conditions such as DR, ODC, glaucoma, or cataract. The Normal class contains 1,293 images from two sources: 669 from RFMiD 1.0 and 624 from OIA-ODIR. The DR class contains 1,098 images and the Glaucoma class contains 701 images, both derived from the original OIH dataset. All newly added samples were screened following the same procedure to ensure no image-level overlap with the pretraining data. The final OIHC dataset comprises four classes: Normal (1,293 images), DR (1,098 images), Glaucoma (701 images), and AMD (505 images). The ODIR-5K dataset is a fundus image dataset released in 2019, containing paired fundus images from 5,000 patients, of which 6,759 images were actually used for pretraining after our screening process. In this study, we adopted a self-supervised contrastive learning framework and pre-trained the model on this dataset, allowing the model to learn general visual representations from fundus images. We then fine-tuned the pre-trained model on OIHC and FIVES to evaluate its generalization performance.

**Table 1:** Data source distribution of the OIHC dataset.

Class	RFMiD 1.0	RFMiD 2.0	OIA-ODIR	OIH	Total
Normal	669	0	624	0	1,293
AMD	155	9	341	0	505
DR	0	0	0	1,098	1,098
Glaucoma	0	0	0	701	701

Fundus images taken in clinics often suffer from poor exposure. Uneven illumination, different equipment, and operator experience are the main causes. This exposure imbalance reduces retinal contrast and blurs critical details such as blood vessels and lesions. As a result, the model faces difficulties in feature extraction and representation learning. Figure 2a presents a low-contrast fundus image sample. To enhance the quality of such samples, we adopt a method called dual-illumination estimation (38) to correct these images. This method performs illumination estimation on both the original image and its inverted version. The former corrects underexposed regions in the original image, whereas the latter addresses overexposed areas. These two estimations yield intermediate images that brighten dark regions and suppress overly bright areas, respectively. Multi-exposure fusion then combines the visually best regions from the two intermediate images and the original input. The final output is an image with uniform global exposure and rich details. Some images contain large irrelevant backgrounds that hinder training efficiency. Therefore, our preprocessing pipeline first removes the black borders around the input image to highlight the foreground and reduce noise. Figure 2b shows the image after border removal. Next, the dual-illumination estimation algorithm is applied to improve visual quality [Figure 2c]. The corrected images achieve balanced global contrast while preserving subtle structures and fine details from the original data. This provides a higher-quality foundation for subsequent feature learning and model analysis. During the supervised learning phase, the corrected images are augmented with techniques such as flipping, rotation, and scaling.

**Figure 2:**
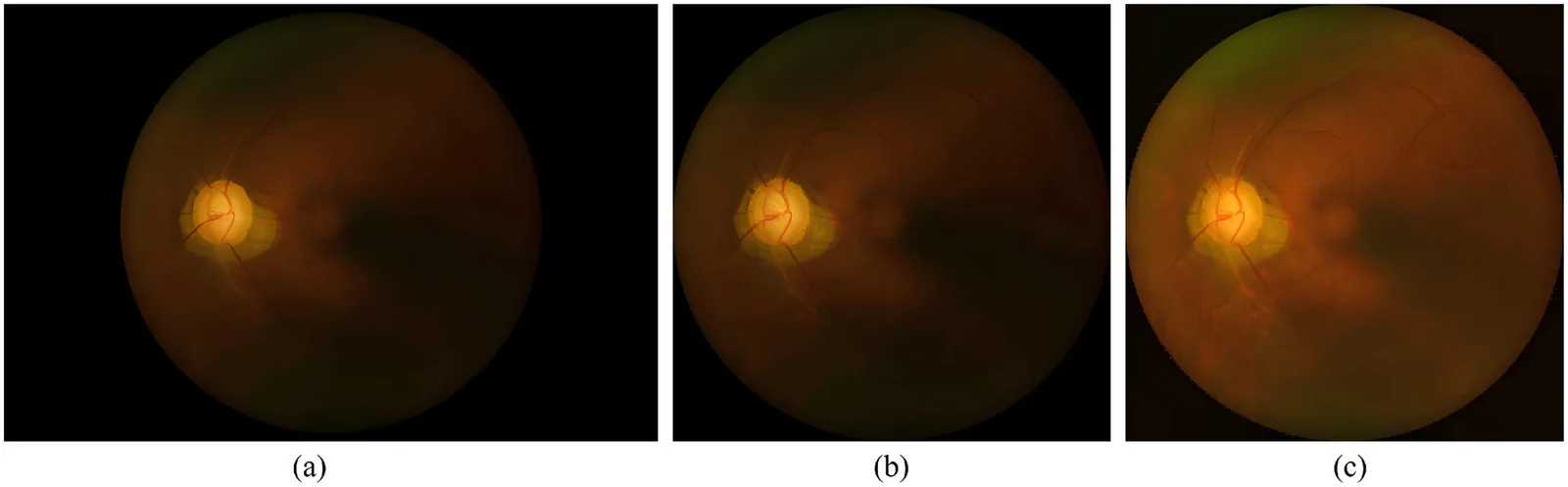
Fundus image preprocessing. **(a)** Original image, **(b)** Removing the black border, and **(c)** Image enhancement using dual-illumination estimation method.

### 3.3 Dual-branch feature extraction architecture

The study proposes a hybrid dual-branch feature extraction architecture consisting of two parallel backbones, EfficientNet-B4 and MambaOut. Both branches are initialized with weights from contrastive self-supervised pre-training. They remain trainable during fine-tuning for the retinal disease classification task. Diagnosing fundus diseases requires identifying local pathological features while also capturing global contextual relationships. Visual Mamba models perform well on long-sequence modeling. However, Mamba's core state space model (SSM) introduces sequential scanning and causal assumptions, which may not suit non-causal vision tasks like image classification. Unlike the original Mamba, MambaOut removes the SSM component, resulting in a simpler design. Research on ImageNet reveals that MambaOut actually achieves better performance after removing the complex SSM module (13). This suggests that the SSM component may introduce unnecessary complexity for image classification tasks. Fundus image classification is a discriminative task based on 2D spatial features, not sequence modeling. Using MambaOut as the feature extraction branch avoids the sequential computation inherent to SSMs. Its lightweight design balances feature depth and breadth under limited computational resources, and its strong modeling ability provides a robust and discriminative feature space for the subsequent classification task. In the training process, these two branches are optimized together. This joint optimization helps mitigate the limitations of single-backbone models and enriches the final feature representation. As a result, the model becomes more accurate and stable for complex fundus disease classification.

### 3.4 Feature-dimensional learnable cross fusion module

The two branches output features with different dimensions. To align them for fusion, we replaced the original FC layers of both the CNN and MambaOut with new ones to match the dimensions. The aligned features then pass through the FDLC module. This component adaptively learns the importance of each branch for different samples and fuses the features selectively. Figure 3 displays the FDLC module. Following this, the two branches output 512-dimensional feature vectors, denoted as $f_{e}$ and $f_{m}$. We concatenate them along the channel dimension to obtain the joint feature $f_{c}=[\;f_{e};f_{m}]\in R^{1024}$. The concatenated feature $f_{c}$ is then fed into two parallel sub-branches for adaptive feature enhancement and dynamic fusion. The left branch is an SE enhancement branch. We adapted the SE block design for high-dimensional feature maps to 1D features. The original SE attention mechanism comprises two steps: squeeze and excitation (39). In the squeeze step, global average pooling compresses the input feature map into a vector, which is then mapped to a smaller vector via a FC layer. However, since our backbone outputs are already 1D vectors, global average pooling is unnecessary. Specifically, $f_{c}$ is first passed through a FC layer to reduce the dimension from 1,024 to 64, using a reduction ratio r=16 to keep parameters low. A ReLU is used to add non-linearity. Then a second FC layer restores the dimension to 1,024, followed by a sigmoid function to normalize outputs to (0,1). The resulting weight is multiplied by $f_{c}$ to get $f_{s}$. This allows the network to selectively enhance or suppress features based on global information, increasing focus on important features. Finally, a linear mapping reduces the dimension to 512, giving $f_{l}\in R^{512}$. The right branch uses a lightweight gating network inside the FDLC module. It adaptively controls how much each branch contributes to the fusion. The gating network takes $f_{c}$ as input. An FC layer maps it to a 256-dimensional space with ReLU activation. Then a second FC layer compresses it into a scalar, followed by a sigmoid activation that normalizes it to (0,1), producing the gating coefficient $gate=\sigma (Linear_{2}(ReLU(Linear_{1}(x))))$. This gating coefficient controls how the two feature paths are fused. When the gate is close to 1, the model prefers the SE-enhanced path. When the gate is close to 0, it keeps more of the original feature $f_{m}$. The final fused feature is calculated as $f_{\;fused}=gate\cdot (\;f_{e}+f_{l})+(1-gate)\cdot f_{m}$. Here, $f_{l}$ is the SE-enhanced feature after linear dimension reduction. This design allows the network to adapt the fusion strategy to the input, improving the flexibility and robustness of feature representation.

**Figure 3:**
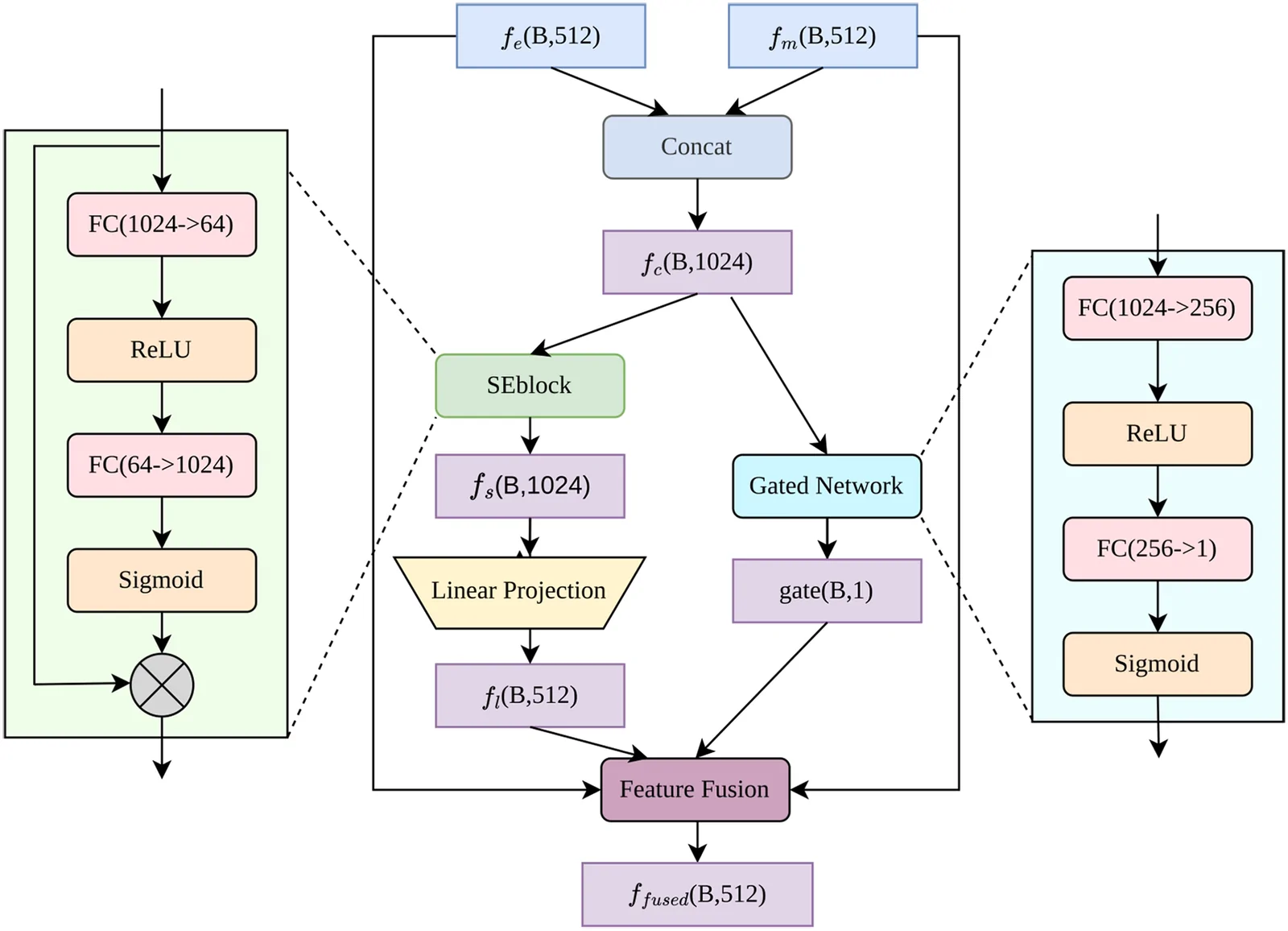
The structure of feature-dimensional learnable cross fusion module.

### 3.5 Self-supervised contrastive pre-training

In the era of deep learning, feature learning has become pivotal for enhancing model performance. This is particularly critical for medical data, where high annotation costs lead to label scarcity despite the abundance of unlabeled data. SimCLR is an early and influential work in this field. It builds on unsupervised learning and introduces contrastive learning. By constructing positive and negative sample pairs, SimCLR learns effective representations from unlabeled data (14). Researchers have applied SimCLR to various tasks, including medical image analysis and semantic segmentation. Our self-supervised contrastive pre-training framework is based on SimCLR to pre-train the dual-branch architecture. This enables the model to learn generalizable representations from unlabeled data for downstream tasks with limited annotations. As shown in Figure 1, a preprocessed input image *x* undergoes two different augmentation strategies (e.g., random cropping, color distortion) to create two views. Each view is then processed by two encoders, EfficientNet-B4 and MambaOut, to extract high-level features, i.e., $h_{effb4}=f_{effb4}(x)$, $h_{mamb}=f_{mamb}(x)$. The features $z_{effb4}=g_{effb4}(h_{effb4})$ and $z_{mamb}=g_{mamb}(h_{mamb})$ are then mapped to a shared latent space through independent non-linear projection heads. Here, g(⋅) is a two-layer MLP. The outputs from the two encoders are fused by the FDLC module. After L2 normalization, a lightweight semantic extractor produces a semantic vector $s=MLP(z_{\;fusion})$ for downstream tasks. For self-supervised pre-training, we use the NT-Xent loss to learn features that are invariant across augmented views of the same image but discriminative between different images. This loss makes the model pull different augmented views of the same image closer in the feature space. It also pushes them away from negative samples including views from other images. So the model learns representations that are robusto perturbations and semantically consistent. During supervised fine-tuning, we load the pre-trained weights from the dual-branch backbone as initialization and apply these weights to all layers except the classifier. With the prior knowledge from pre-training, the model adapts to downstream medical diagnosis using limited labeled samples. Moreover, we fine-tune the whole model using focal loss, along with learning rate scheduling and regularization to prevent overfitting in few-shot scenarios. This alleviates the reliance on large annotated datasets while the pre-trained model learns to capture rich semantic representations from medical images. It is meaningful for clinical settings with limited annotation resources.

### 3.6 Classifier module

During fine-tuning, we replace the standard linear classification head with the dendritic classifier. Biological studies show that neurons do not simply aggregate all input signals. Instead, their dendritic structures can perform local non-linear computations on synaptic inputs. This enhances the neuron's representational capacity and pattern recognition accuracy. Based on this theory, Xu et al. (16) designed a dendritic neuron-based multilayer perceptron and provided its implementation. In this work, we adapt this classifier for a dual-branch network focused on fundus image classification. It learns more discriminative high-order features, improving classification performance for various fundus diseases. This module derives from a dendritic neural unit, which divides a neuron's decision process into four stages: synaptic, dendritic, membrane, and soma layers. Figure 4 shows the structure of dendritic neural unit.

**Figure 4:**
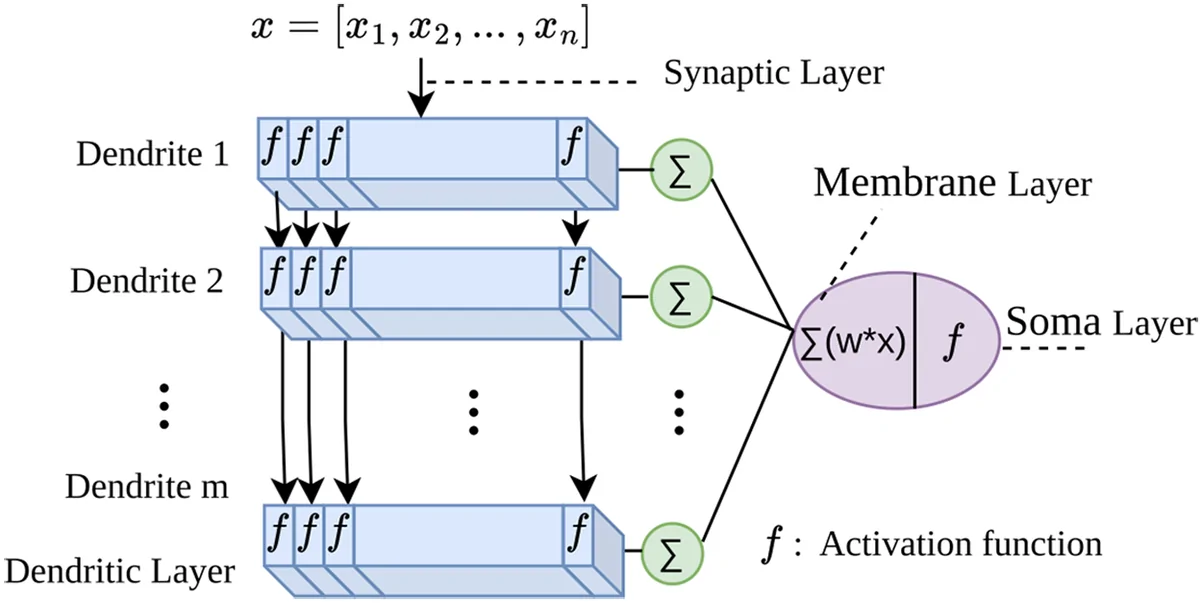
The structure of dendritic neural unit.

Let *x* be the input features from the dual-branch network after feature extraction and fusion. The input first goes to thedth synaptic layer, which models how synapses modulate signals. For our multi-class task, we set *K* dendritic neurons, one for each disease category. Then, for the ith synapse on the dendritic branch of the kth dendritic neuron, we first apply a linear transformation to x as defined in Equation (1).

$$S_{id}^{k}=w_{id}^{k}\cdot LayerNorm(x_{id}^{k})+b_{id}^{k}$$ (1)

where $x_{id}^{k}$ is the input to the ith synapse on the dth dendritic branch. $w_{id}^{k}$ is the synaptic weight, representing how the synapse enhances or inhibits the signal. $b_{id}^{k}$ is a bias, modeling the threshold needed for the postsynaptic membrane to generate an effective potential. $S_{id}^{k}$ is the output of that synapse. This layer lets the model assign different initial attention to various fundus pathological features. The dendritic layer integrates synaptic inputs from the same branch. It applies a non-linear activation to aggregate signals. This allows each dendritic branch to learn specific patterns of pathological features. The output $D_{d}^{k}$ of the dth branch in the kth dendritic unit is calculated using Equation (2).

$$D_{d}^{k}=\sum_{i=1}^{n}LayerNorm(\psi (S_{id}^{k}))$$ (2)

Here, *n* is the number of synapses per dendritic layer, and ψ(⋅) is the activation function for that layer. We use the sigmoid function to map local signals non-linearly to (0,1). Thus, different dendritic branches learn different feature combinations. The membrane layer then sums the outputs from all branches with weights, simulating signal convergence onto the soma membrane. Finally, the soma layer gives the output of each dendritic unit as defined in Equation (3).

$$y^{k}=\sum_{d=1}^{m}D_{d}^{k},Y=[y^{1},y^{2},\cdots ,y^{K}]$$ (3)

Here, *m* is the number of dendritic layers, and k=1,2,⋯,K.

## 4 Results and discussion

### 4.1 Experimental setup

#### 4.1.1 Evaluation metrics

Accuracy (Acc), Precision (Pre), Recall (Rec), F1-score, and Kappa are used to evaluate model performance. Specifically, accuracy measures the overall correctness, i.e., the proportion of correctly classified samples. Precision focuses on the reliability of positive predictions, which is the ratio of true positives to all predicted positives. In contrast, recall reflects the coverage of actual positives, defined as the ratio of true positives to all ground-truth positives. The F1-score balances precision and recall through their harmonic mean. A high F1-score indicates a good balance between them, thus offering a more robust evaluation for the positive class. The Kappa score assesses the agreement between predictions and ground truth while correcting for chance. A value above 0.8 is commonly considered substantial agreement. The specific formulas for these metrics are given in Equations (4)-(8).

$$Acc=\frac{TP+TN}{TP+FP+TN+FN}$$ (4)

$$Pre=\frac{TP}{TP+FP}$$ (5)

$$Rec=\frac{TP}{TP+FN}$$ (6)

$$F1-score=\frac{2*Pre*Rec}{Pre+Rec}$$ (7)

$$Kappa=\frac{P_{o}-P_{e}}{1-P_{e}},P_{o}=\frac{1}{N}\sum_{i=1}^{K}n_{ii},P_{e}=\sum_{i=1}^{K}\left(\frac{R_{i}}{N}\cdot \frac{S_{i}}{N}\right)$$ (8)

where TP denotes the number of samples correctly predicted as positive. TN denotes the number correctly predicted as negative. FP denotes the number of negative samples wrongly classified as positive. FN denotes the number of positive samples wrongly classified as negative. $P_{o}$ is the proportion of actual agreement between predictions and ground truth. $P_{e}$ is the probability of chance agreement under random prediction. *K* is the number of categories. $n_{ii}$ is the diagonal element of the confusion matrix, representing the number of correctly classified samples for the *i*th class. $R_{i}$ is the number of ground truth samples for the *i*th class. $S_{i}$ is the number of predicted samples for the *i*th class. *N* is the total number of samples.

#### 4.1.2 Implementation details

The experiments in this study were conducted in a development environment equipped with a 14 vCPU Intel(R) Xeon(R) Gold 6,330 CPU @ 2.00 GHz, 90GB of RAM, and an RTX 3,090 GPU (24 GB). The software stack was based on Python 3.10 and PyTorch 2.1.2 with CUDA 11.8, running on the Ubuntu 22.04 operating system. Since the raw images were high-resolution with varying dimensions, all images were resized to 224 × 224 pixels to ensure training consistency. Pre-training was conducted with a batch size of 200 and a total of 100 epochs. Model training employed the AdamW optimizer with a batch size of 16, an initial learning rate of 1e-3, and a weight decay of 0.01. The learning rate was updated using the ExponentialLR scheduler with a gamma value of 0.9. To avoid data leakage and ensure the rigor of our experiments, we adopted a five-fold cross-validation strategy. The splitting method varied across datasets depending on the available information. For the OIA-ODIR subset within OIHC, the official release provides patient IDs and left/right eye mappings. We performed strict patient-level splitting accordingly, ensuring that the left and right eye images of the same patient were not split between training and testing. For the RFMiD subset and the Glaucoma category, no official patient mapping is available. Therefore, image-level splitting was applied. As for the DR category retained from the original OIH dataset, images were grouped by the numeric prefix and left/right eye identifiers in the filenames. This grouping is inference-based and cannot be fully validated due to the lack of an official patient mapping file in OIH. Regarding the FIVES dataset, no patient information is available for reference, so we grouped images by their numeric filename prefix, assigning images sharing the same prefix to the same fold. These are conservative splitting approaches we adopted under the data constraints, rather than strict patient-level splits. For all datasets, stratified sampling was applied within each fold during the five-fold splitting to preserve the class distribution. The final results are reported as the mean and standard deviation across the five folds. For model training, we adopted Focal Loss as the classification loss function. It includes two core mechanisms. The class weight parameter alleviates the bias caused by class imbalance. The focusing parameter reduces the loss contribution from easy-to-classify samples and encourages the model to focus on hard samples near the decision boundary (40). In the OIHC dataset, the class distribution is Normal 1,293, DR 1,098, Glaucoma 701, and AMD 505, indicating a certain degree of imbalance. We therefore used the weight mechanism of Focal Loss to mitigate the bias toward majority classes. For the FIVES dataset, the class distribution is relatively balanced across the original dataset, though 5-fold cross-validation introduces slight fluctuations in class proportions across folds. In this case, the focusing parameter of Focal Loss also helps improve the model's ability to discriminate ambiguous and atypical cases in fundus disease classification.

### 4.2 Performance assessment of different backbones

To verify the classification performance of different backbones on the OIHC and FIVES datasets, we evaluated seven mainstream backbones. They are ResNet34, DenseNet121, MobileNet, InceptionNet, ViT, MambaOut, and EfficientNet-B4. The metrics include Accuracy, Precision, Recall, F1-score, and Kappa. Table 2 reports the F1-score and Kappa values of different backbones on the OIHC and FIVES datasets. On OIHC, MambaOut achieved an F1-score of 94.61 ± 0.31% and a Kappa of 93.79 ± 0.63%. EfficientNet-B4 ranked second, with scores of 94.42 ± 0.34% and 93.58 ± 0.55%, followed by InceptionNet. On the FIVES dataset, the top three networks were MambaOut, EfficientNet-B4, and ResNet34. MambaOut and EfficientNet-B4 showed nearly equivalent performance, while EfficientNet-B4 performed slightly better than ResNet34. Although MambaOut achieved the highest overall performance among the evaluated backbones, its parameter count is larger than that of EfficientNet-B4. To balance efficiency and performance, we selected EfficientNet-B4 and MambaOut as the two branches of our dual-branch architecture. Specifically, EfficientNet-B4 serves as a lightweight yet powerful CNN branch to capture fine-grained local features. MambaOut provides wider spatial coverage through its large-kernel design and feature refinement. This combination leverages the strengths of both architectures while maintaining a reasonable parameter budget. The fused features provide richer representations for the downstream classification task.

**Table 2:** 5-fold cross-validation results of different backbones on the OIHC and FIVES datasets.

Backbone	OIHC		FIVES	
	F1-score (%)	Kappa (%)	F1-score (%)	Kappa (%)
DenseNet121	93.99 ± 0.49	92.94 ± 0.55	93.12 ± 0.42	90.82 ± 0.60
MobileNet-V3	93.83 ± 0.51	92.67 ± 0.78	92.89 ± 0.85	90.51 ± 1.13
InceptionNet	94.24 ± 0.41	93.31 ± 0.37	93.21 ± 0.58	91.02 ± 0.71
ViT	94.03 ± 0.34	93.16 ± 0.46	91.76 ± 1.93	89.00 ± 2.60
ResNet34	94.19 ± 0.45	93.49 ± 0.67	93.50 ± 0.74	91.33 ± 0.95
EfficientNet-B4	94.42 ± 0.34	93.58 ± 0.55	93.61 ± 0.27	91.45 ± 0.34
MambaOut	94.61 ± 0.31	93.79 ± 0.63	93.64 ± 0.26	91.50 ± 0.38

### 4.3 Ablation study

In this section, we present ablation studies to evaluate our dual-branch system. We gradually add key fusion components and calculate the metrics. Model1 serves as the baseline, using a single EfficientNet-B4 for feature extraction. Adding a MambaOut branch yields Model2, which forms a dual-branch architecture with fixed linear weights (both set to 0.5) for branch fusion. Model3 retains the same dual-branch structure but replaces the fixed weights with learnable dynamic weights. Model4 further replaces the dynamic weighting with the proposed FDLC mechanism. Model5 introduces contrastive learning pre-training, initializing the dual-branch network with weights learned from unlabeled data. All five models described above use a traditional linear classifier. Finally, Model6 builds on Model5 but replaces the linear classifier with a dendritic classifier.

Table 3 shows the ablation results on the OIHC and FIVES datasets. The performance improvements are consistent from Model1 to Model6 on both datasets. On the OIHC dataset, the baseline model achieves an accuracy of 95.39%, an F1-score of 94.42%, and a Kappa of 93.58%. After incorporating the MambaOut branch with fixed-weight fusion, the three metrics improve to 95.94%, 95.01%, and 94.36%, respectively. Replacing the fixed weights with learnable dynamic weights further raises the performance to 96.08%, 95.21%, and 94.56%. The introduction of the FDLC fusion module brings additional gains, reaching 96.36%, 95.50%, and 94.93%. After incorporating SimCLR pre-training, the model achieves 96.78%, 96.05%, and 95.51%. Finally, replacing the linear classifier with the dendritic classifier yields the best overall performance, with the three metrics reaching 97.03%, 96.32%, and 95.89%, respectively. The same trend is consistently observed on the FIVES dataset, where Model 6 achieves the highest F1-score of 95.10% and Kappa of 93.49%, supporting the effectiveness of each proposed component. To assess whether these differences are statistically meaningful, we performed paired t-tests on the fold-wise F1-score differences between consecutive model configurations. The results in Table 4 show that the four selected comparisons yield p-values below 0.01, and these results remain below the Bonferroni-corrected threshold (*α* = 0.0125). These findings suggest that the observed improvements are unlikely to be attributable to random fluctuation.

**Table 3:** 5-fold cross-validation results of the ablation study on the OIHC and FIVES datasets.

Models	Description	Dataset	Acc (%)	F1-score (%)	Kappa (%)
Model 1	Baseline	OIHC	95.39 ± 0.43	94.42 ± 0.34	93.58 ± 0.55
		FIVES	93.62 ± 0.27	93.61 ± 0.27	91.45 ± 0.34
Model 2	Baseline + MambaOut + fixed weights	OIHC	95.94 ± 0.20	95.01 ± 0.32	94.36 ± 0.29
		FIVES	93.98 ± 0.30	93.96 ± 0.32	91.99 ± 0.41
Model 3	Baseline + MambaOut + dynamic weights	OIHC	96.08 ± 0.25	95.21 ± 0.35	94.56 ± 0.36
		FIVES	94.12 ± 0.31	94.11 ± 0.34	92.18 ± 0.30
Model 4	Baseline + MambaOut + FDLC	OIHC	96.36 ± 0.30	95.50 ± 0.32	94.93 ± 0.41
		FIVES	94.34 ± 0.40	94.31 ± 0.41	92.37 ± 0.39
Model 5	Baseline + MambaOut + FDLC + SimCLR	OIHC	96.78 ± 0.27	96.05 ± 0.33	95.51 ± 0.37
		FIVES	94.82 ± 0.46	94.78 ± 0.46	92.87 ± 0.49
Model 6	Baseline + MambaOut + FDLC + SimCLR + dendritic classifier	OIHC	97.03 ± 0.31	96.32 ± 0.38	95.89 ± 0.43
		FIVES	95.13 ± 0.53	95.10 ± 0.54	93.49 ± 0.71

**Table 4:** Paired *t*-test *p*-values for F1-score comparisons between different model configurations on the OIHC and FIVES datasets.

Models	OIHC	FIVES
Model 1 vs. Model 2	0.0083	0.0033
Model 3 vs. Model 4	0.0016	0.0064
Model 4 vs. Model 5	0.0017	<0.001
Model 5 vs. Model 6	0.0014	0.0013

The *p*-values shown in the table are uncorrected values from paired t-tests. The significance threshold after Bonferroni correction for multiple comparisons (*α* = 0.05/4 = 0.0125) is provided for reference.

We further investigated the impact of data preprocessing and input resolution on model performance. As shown in Table 5, applying dual-illumination preprocessing improved the F1-score from 95.13% to 96.32% on OIHC and from 94.38% to 95.10% on FIVES, indicating that this preprocessing step effectively enhances image quality and benefits subsequent classification. We also compared two input resolutions, 224 × 224 and 384 × 384, on both datasets. Increasing the resolution from 224 to 384 led to a modest improvement in F1-score, with gains of approximately 0.15 percentage points on OIHC and 0.27 percentage points on FIVES. However, this improvement was accompanied by a notable increase in computational cost, with FLOPs rising from 6.06G to 17.79G, representing an approximately 2.9-fold increase. Considering the trade-off between the limited performance gain and the increased computational overhead, we selected 224 × 224 as the primary input resolution for our experiments. This choice maintains competitive performance while keeping training and inference costs manageable.

**Table 5:** Effects of dual-illumination preprocessing and input resolution on model performance.

Models	Dataset	Acc (%)	Pre(%)	Rec (%)	F1-score (%)	Kappa (%)
w/o dual-illumination	OIHC	96.01 ± 0.22	95.18 ± 0.49	95.15 ± 0.60	95.13 ± 0.35	94.47 ± 0.34
	FIVES	94.37 ± 0.44	94.59 ± 0.24	94.35 ± 0.57	94.38 ± 0.44	92.49 ± 0.60
Input size 224 × 224	OIHC	97.03 ± 0.31	96.54 ± 0.57	96.12 ± 0.26	96.32 ± 0.38	95.89 ± 0.43
	FIVES	95.13 ± 0.53	95.19 ± 0.49	95.11 ± 0.61	95.10 ± 0.54	93.49 ± 0.71
Input size 384 × 384	OIHC	97.14 ± 0.35	96.63 ± 0.91	96.39 ± 0.29	96.47 ± 0.35	96.05 ± 0.48
	FIVES	95.38 ± 0.35	95.44 ± 0.34	95.40 ± 0.39	95.37 ± 0.38	93.83 ± 0.46

We also evaluated the contribution of SSL pre-training under limited annotation scenarios. Model5 is built upon Model4 with SimCLR pre-training. We compared the two models using three labeling ratios of 25%, 50%, and 100% of the training data, as shown in Figure 5. On the OIHC dataset, with 100% training labels, SSL pre-training improved the F1-score from 95.50% to 96.05%, a gain of 0.55 percentage points. When the label ratio was reduced to 50%, the performance improved from 93.39% to 94.31%, a gain of 0.92 percentage points. With only 25% of the labels available, the pre-trained model achieved 91.81% compared to 90.47% for the baseline without SSL, a gain of 1.34 percentage points. A similar trend was observed on the FIVES dataset. SSL pre-training improved the performance from 94.31% to 94.78% at 100% labels, a gain of 0.47 percentage points; from 90.44% to 91.42% at 50% labels, a gain of 0.98 percentage points; and from 85.04% to 86.62% at 25% labels, a gain of 1.58 percentage points. These results indicate that SSL pre-training provides consistent improvements across different label ratios, with larger gains observed when labeled data is scarce. In both datasets, the largest gains were observed under the 25% label condition.

**Figure 5:**
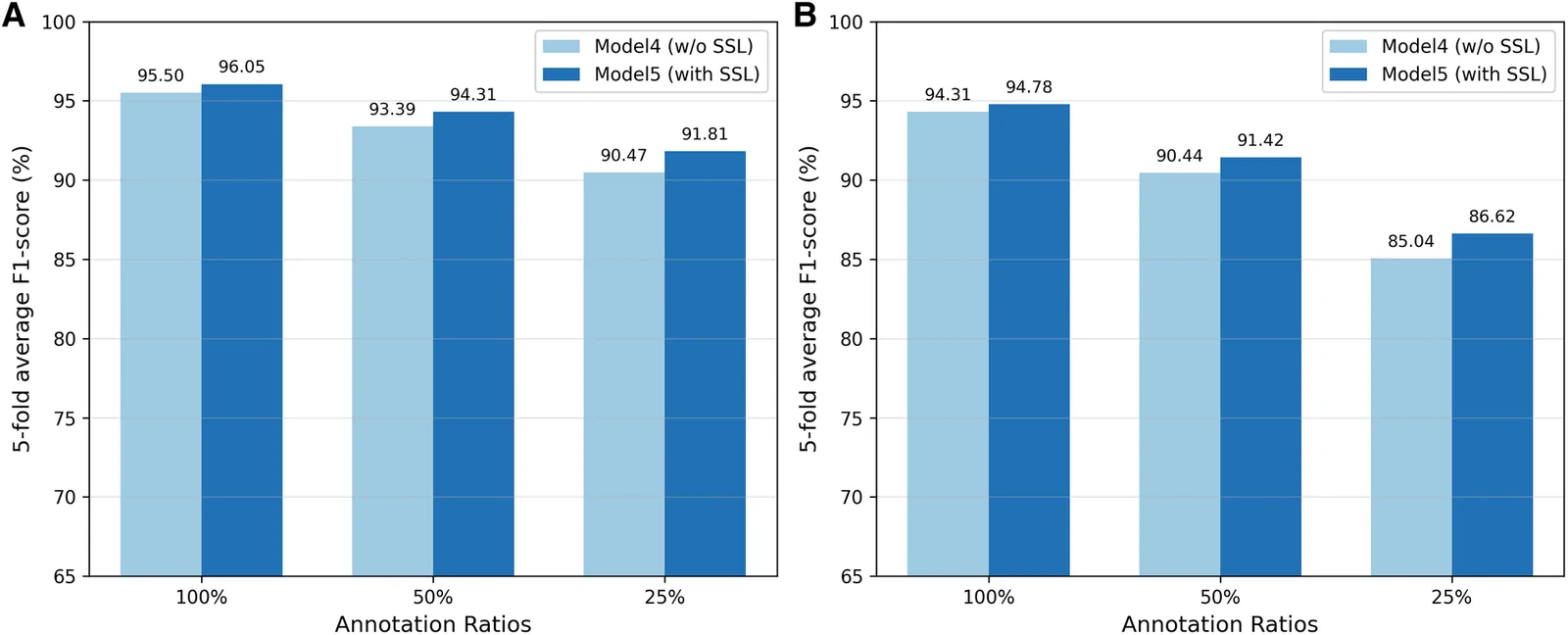
5-fold average F1-score of Model4 and Model5 under three labeling ratios on the OIHC and FIVES datasets. **(a)** OIHC, **(b)** FIVES.

To further investigate the effectiveness of the proposed modules, we used t-SNE to project the feature space of model2 and model6 onto a 2D plane. As illustrated in Figure 6a, the feature space of Model2 shows partial overlap among the four classes. The Normal and AMD samples overlap in the central-lower region. The DR samples are scattered across two clusters on the left side, with some overlapping with the Normal class. The Glaucoma class forms two separate clusters in the upper region, but their separation from the other classes is not clear. In contrast, as shown in Figure 6b, the feature space of Model6 shows more compact clustering and clearer inter-class separation. The Normal and AMD classes are now better separated, with the AMD samples forming a tighter cluster in the upper-left region. The DR samples are grouped into a more compact cluster on the right side with less dispersion. The Glaucoma class remains as a distinct cluster in the upper-right region. These results indicate that the proposed modules improve both intra-class compactness and inter-class separability of the learned feature representations.

**Figure 6:**
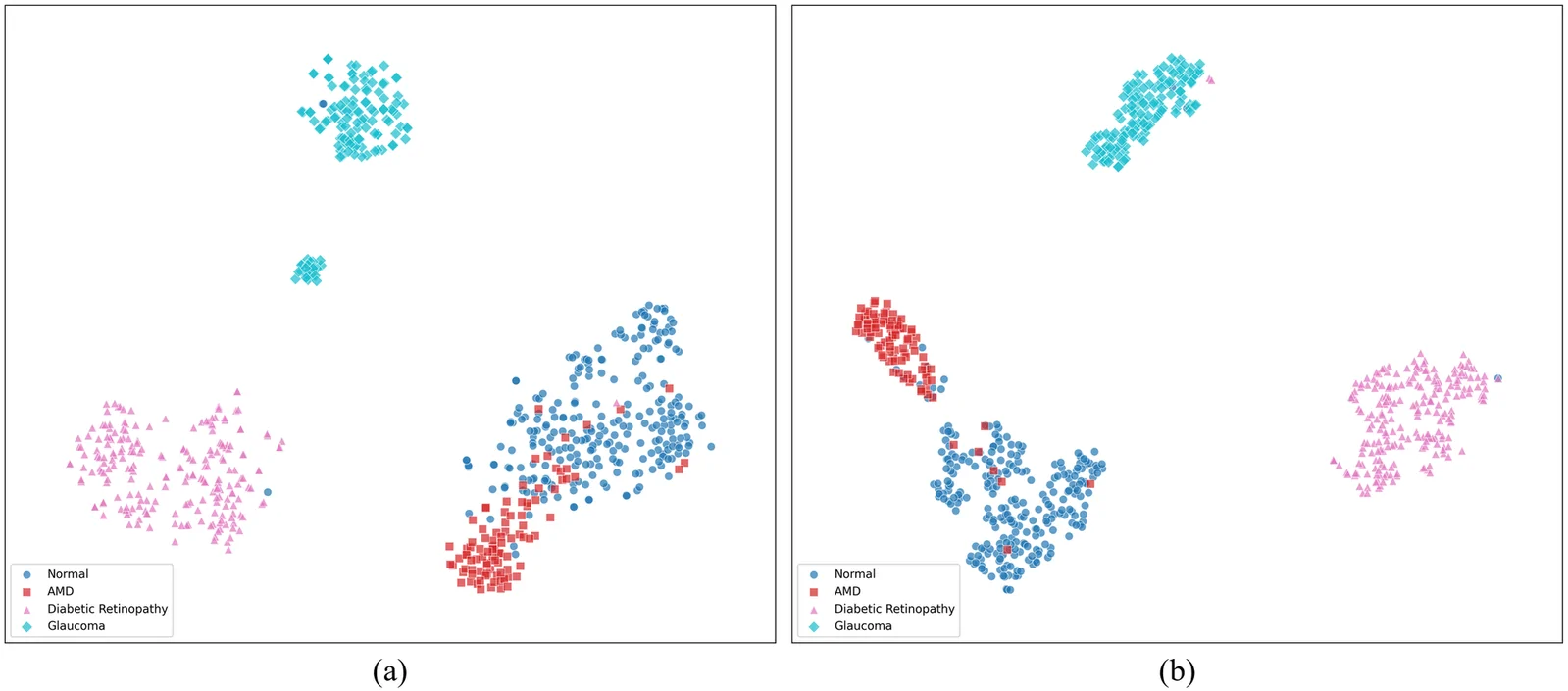
t-SNE visualization of Model 2 and Model 6 on the OIHC dataset. **(a)** Model 2, **(b)** Model6.

### 4.4 Comparison experiments

To further benchmark the performance of the proposed model, we evaluated several state-of-the-art algorithms on both the OIHC and FIVES datasets. The selected models include HRNet (41), Swin-T (42), ConvNeXt (43), MambaOut (13), Dense-XGB (23), Iwin-B (44), and DSA-mamba (45). The performance metrics employed are consistent with those mentioned above. All baseline models were re-implemented under the same data splitting and training configurations to ensure fair comparison. Table 6 presents the detailed experimental results of the comparative methods on the OIHC and FIVES datasets. The results indicate that the proposed model achieves competitive performance across all evaluation metrics. On the OIHC dataset, our model achieves the highest performance among all compared methods, with an accuracy of 97.03 ± 0.31%, an F1-score of 96.32 ± 0.38%, and a Kappa of 95.89 ± 0.43%. Compared to the strongest baseline DSA-mamba, our model brings improvements of 0.70 percentage points in accuracy and 0.98 percentage points in Kappa. On the FIVES dataset, our model also achieves the highest accuracy of 95.13 ± 0.53% and F1-score of 95.10 ± 0.54%, with improvements of 1.11 and 1.05 percentage points over DSA-mamba, respectively. To assess whether these differences are statistically meaningful, we performed paired t-tests between the proposed model and each baseline using the five-fold cross-validation results. As shown in Table 7, these comparisons yield p-values below 0.01, suggesting that the observed improvements are unlikely to be due to random fluctuation. We also calculated the 95% confidence intervals (CI) for each metric, which are reported in Table 6 alongside the means and standard deviations. Excluding HRNet, which yielded noticeably lower performance than the other baselines, the F1-score gains of the proposed model over the remaining methods ranged from 0.72 to 1.71 percentage points on the OIHC dataset, and from 1.05 to 2.27 percentage points on the FIVES dataset. While these gains are relatively modest in absolute terms, they are consistent across both datasets and all evaluation metrics, supporting the effectiveness of the proposed dual-branch design. Table 8 summarizes the computational efficiency of all compared models in terms of parameter count, FLOPs, and inference time.

**Table 6:** 5-fold cross-validation results of different models on OIHC and FIVES (95% CI in brackets).

Models	Dataset	Acc (%)	Pre (%)	Rec (%)	F1-score (%)	Kappa (%)
HRNet (41)	OIHC	91.88 ± 1.34 [90.22, 93.54]	91.60 ± 2.38 [88.64, 94.55]	89.08 ± 2.06 [86.52, 91.63]	89.84 ± 1.85 [87.54, 92.14]	88.64 ± 1.86 [86.33, 90.95]
	FIVES	88.59 ± 2.31 [85.72, 91.46]	89.23 ± 2.27 [86.42, 92.05]	88.62 ± 2.36 [85.69, 91.55]	88.63 ± 2.28 [85.80, 91.45]	84.83 ± 3.14 [80.94, 88.73]
Swin-T (42)	OIHC	96.00 ± 0.34 [95.57, 96.42]	95.46 ± 1.18 [94.00, 96.92]	95.21 ± 1.31 [93.59, 96.83]	95.18 ± 0.46 [94.61, 95.75]	94.44 ± 0.48 [93.85, 95.03]
	FIVES	93.52 ± 0.70 [92.65, 94.39]	93.68 ± 0.68 [92.84, 94.52]	93.49 ± 0.72 [92.60, 94.39]	93.50 ± 0.71 [92.62, 94.38]	91.33 ± 0.95 [90.15, 92.52]
ConvNeXt (43)	OIHC	96.27 ± 0.40 [95.77, 96.77]	95.87 ± 0.87 [94.80, 96.95]	95.31 ± 0.61 [94.55, 96.06]	95.54 ± 0.50 [94.92, 96.16]	94.82 ± 0.55 [94.14, 95.50]
	FIVES	93.38 ± 0.56 [92.68, 94.08]	93.68 ± 0.47 [93.10, 94.26]	93.36 ± 0.52 [92.71, 94.01]	93.39 ± 0.55 [92.70, 94.07]	91.17 ± 0.75 [90.24, 92.10]
MambaOut (13)	OIHC	95.53 ± 0.49 [94.92, 96.13]	94.99 ± 1.69 [92.90, 97.09]	94.66 ± 1.09 [93.31, 96.02]	94.61 ± 0.31 [94.22, 95.00]	93.79 ± 0.63 [93.01, 94.56]
	FIVES	93.63 ± 0.28 [93.28, 93.98]	93.77 ± 0.28 [93.42, 94.12]	93.62 ± 0.27 [93.28, 93.97]	93.64 ± 0.26 [93.32, 93.96]	91.50 ± 0.38 [91.04, 91.97]
Dense-XGB (23)	OIHC	96.11 ± 0.43 [95.58, 96.64]	95.89 ± 0.84 [94.84, 96.94]	94.69 ± 0.52 [94.04, 95.34]	95.21 ± 0.47 [94.63, 95.79]	94.58 ± 0.59 [93.84, 95.31]
	FIVES	94.02 ± 0.36 [93.58, 94.47]	94.16 ± 0.32 [93.76, 94.55]	94.00 ± 0.34 [93.57, 94.43]	93.98 ± 0.33 [93.57, 94.39]	92.00 ± 0.45 [91.44, 92.57]
Iwin-B (44)	OIHC	95.83 ± 0.69 [94.97, 96.69]	95.29 ± 1.52 [93.41, 97.17]	94.95 ± 0.89 [93.85, 96.06]	95.02 ± 0.75 [94.09, 95.96]	94.20 ± 0.95 [93.03, 95.38]
	FIVES	92.87 ± 0.72 [91.99, 93.76]	93.21 ± 0.71 [92.32, 94.11]	92.86 ± 0.73 [91.95, 93.77]	92.83 ± 0.77 [91.87, 93.79]	90.50 ± 0.95 [89.32, 91.68]
DSA-mamba (45)	OIHC	96.33 ± 0.37 [95.86, 96.79]	95.58 ± 0.66 [94.75, 96.40]	95.68 ± 0.62 [94.91, 96.46]	95.60 ± 0.44 [95.05, 96.14]	94.91 ± 0.52 [94.26, 95.55]
	FIVES	94.02 ± 0.55 [93.34, 94.70]	94.17 ± 0.29 [93.81, 94.54]	94.01 ± 0.56 [93.31, 94.69]	94.05 ± 0.51 [93.36, 94.64]	92.00 ± 0.74 [91.07, 92.92]
Proposed	OIHC	97.03 ± 0.31 [96.65, 97.42]	96.54 ± 0.57 [95.83, 97.26]	96.12 ± 0.26 [95.79, 96.45]	96.32 ± 0.38 [95.85, 96.78]	95.89 ± 0.43 [95.35, 96.43]
	FIVES	95.13 ± 0.53 [94.47, 95.78]	95.19 ± 0.49 [94.57, 95.80]	95.11 ± 0.61 [94.36, 95.87]	95.10 ± 0.54 [94.42, 95.77]	93.49 ± 0.71 [92.62, 94.37]

**Table 7:** Paired t-test p-values for F1-score comparisons between the proposed model and baseline methods on the OIHC and FIVES datasets.

Proposed vs.	OIHC	FIVES
Swin-T (42)	0.0034	0.0006
ConvNeXt (43)	0.0084	0.0059
Dense-XGB (23)	0.0018	0.0091
Iwin-B (44)	0.0099	0.0014
DSA-mamba (45)	0.0052	0.0012

The *p*-values shown in the table are uncorrected values from paired t-tests. The significance threshold after Bonferroni correction for multiple comparisons (*α* = 0.05/5 = 0.01) is provided for reference.

**Table 8:** Computational efficiency comparison of different models.

Models	Params (M)	FLOPs (G)	Inference time (ms)
HRNet (41)	40.37	9.01	26.39
Swin-T (42)	27.52	2.98	14.10
ConvNeXt (43)	87.57	15.37	14.46
MambaOut (13)	24.25	4.48	7.10
Dense-XGB (23)	13.91	11.58	10.97
Iwin-B (44)	90.22	15.57	18.82
DSA-mamba (45)	35.93	5.64	17.51
Proposed	43.94	6.06	17.73

Figures 7a, 7b show the confusion matrices of the proposed model on the OIHC and FIVES datasets, with values row-normalized to indicate the percentage of samples from each true class. On the OIHC dataset, the model achieves high classification accuracy for the Normal, DR, and Glaucoma categories, at 96.6%, 99.7%, and 99.6%, respectively, while the accuracy for the AMD category is 88.6%. The primary misclassification occurs between AMD and Normal, with approximately 11.0% of AMD samples being misclassified as Normal. This is consistent with the clinical challenge of distinguishing early AMD from age-related normal changes. In addition, the AMD samples in the OIHC dataset originate from two different source datasets, RFMiD and OIA-ODIR, which differ in their annotation strategies, image acquisition conditions, and disease definition criteria. Such differences may lead the model to learn inconsistent feature representations from AMD samples of different origins. Heterogeneity in image quality and imaging conditions may also increase the difficulty of learning discriminative features for the AMD category, thereby affecting classification accuracy to some extent. On the FIVES dataset, the model achieves diagonal accuracies of 97.0% for Normal, 93.9% for AMD, 93.6% for DR, and 96.0% for Glaucoma. The experimental results based on 5-fold cross-validation show that the proposed model achieves the highest macro-average AUC on both datasets, with 0.9939 ± 0.0020 on OIHC and 0.9891 ± 0.0026 on FIVES. The EfficientNet-B4 single-branch model achieved 0.9814 ± 0.0050 on OIHC and 0.9757 ± 0.0052 on FIVES, while the MambaOut single-branch model achieved 0.9829 ± 0.0034 on OIHC and 0.9758 ± 0.0056 on FIVES. Figures 7c, 7d present the ROC curves of the single-branch models and the dual-branch fusion model on a representative fold of each dataset (the values shown in the figures correspond to this representative fold). These results further indicate that the fusion strategy effectively integrates features from the two branches and contributes to improved classification performance.

**Figure 7:**
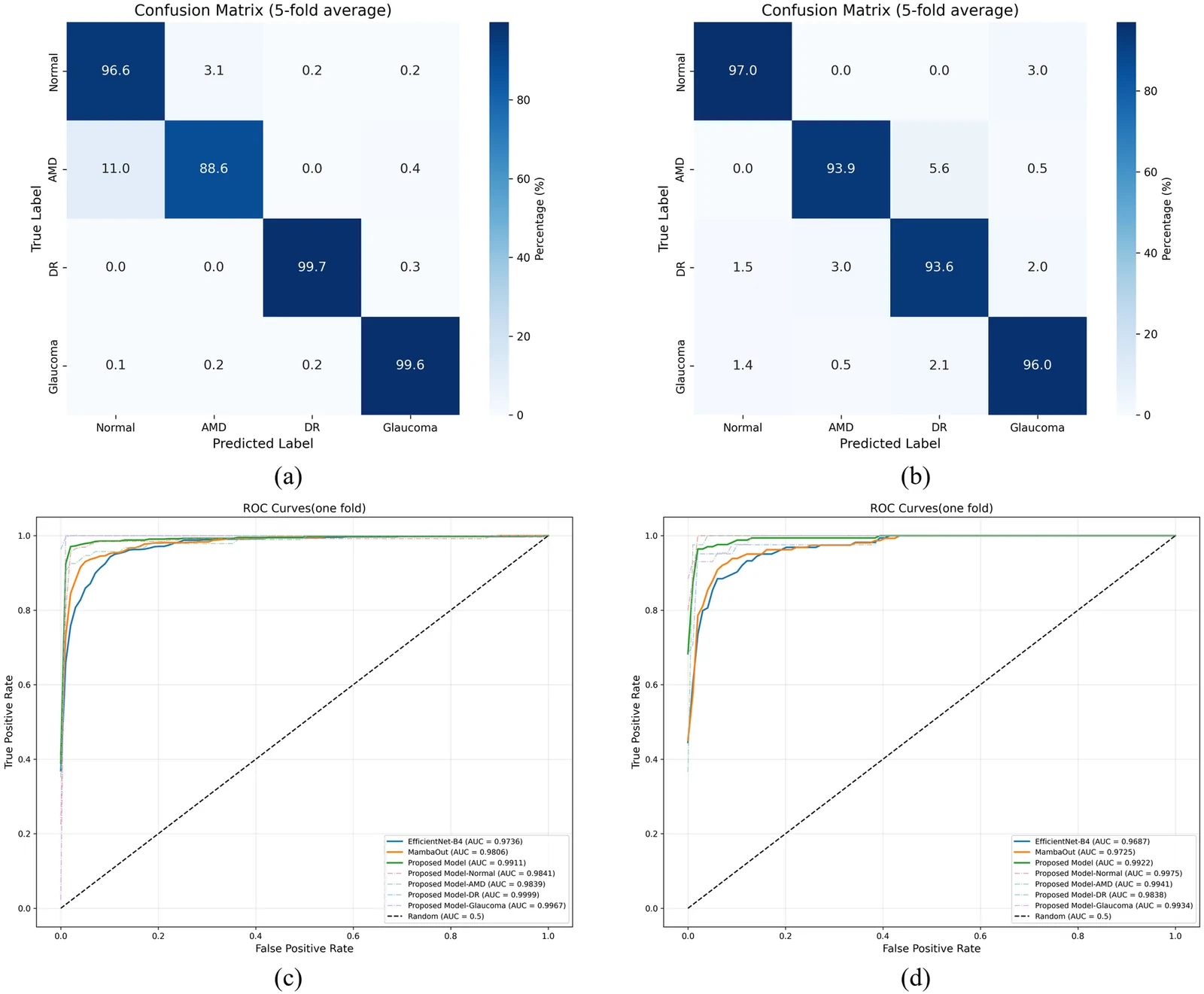
Confusion matrices and ROC curves of the proposed model. **(a)** 5-fold average confusion matrix on OIHC, **(b)** 5-fold average confusion matrix on FIVES, **(c)** ROC curves of the single-branch models (EfficientNet-B4 and MambaOut) and the proposed model on a representative OIHC fold, and **(d)** ROC curves of the single-branch models (EfficientNet-B4 and MambaOut) and the proposed model on a representative FIVES fold.

### 4.5 Robustness evaluation

To evaluate the stability of the proposed model under image quality degradation, we applied five types of distortions to the test set: Gaussian noise, salt-and-pepper noise, Gaussian blur, brightness change, and JPEG compression. The parameters for each distortion were set as follows: Gaussian noise with *σ* = 0.03, salt-and-pepper noise with a density of 0.02, Gaussian blur with a kernel size of 5 × 5 and *σ* = 1.0, brightness change with a scaling factor of 0.6 (reducing image brightness to 60% of the original), and JPEG compression with a quality factor of 80 (where 100 indicates no compression and lower values indicate higher compression). The same degraded images were used for all models to ensure fair comparison. Table 9 and Figure 8 present the 5-fold average F1-score results and performance trends of the robustness evaluation on the OIHC and FIVES datasets. As reported in Table 9, our model achieves the highest F1-score across all six conditions on both datasets, with a relatively stable decline. HRNet consistently exhibits the lowest F1-score across all degradation types on both datasets. DSA-mamba and MambaOut are among the top-performing models, with DSA-mamba generally ranking second behind our model. According to Figures 8a, 8b, our model exhibits the least fluctuation across all degradation types, whereas the other models all experience substantial performance degradation under certain conditions. The performance gap over the DSA-mamba method widens under JPEG compression on OIHC and under salt-and-pepper noise on FIVES. Swin-T drops considerably under JPEG compression, and Dense-XGB shows a notable decline under salt-and-pepper noise and JPEG compression.

**Table 9:** Comparison of 5-fold averaged F1-score of different models under various image degradations on the OIHC and FIVES datasets.

Models	Dataset	Clean (%)	Gaussian noise (%)	Salt-Pepper noise (%)	Gaussian blur (%)	Brightness change (%)	JPEG compression (%)
HRNet (41)	OIHC	89.84	84.15	82.67	87.01	79.84	87.73
	FIVES	88.63	83.22	81.56	84.53	81.21	85.10
Swin-T (42)	OIHC	95.18	92.57	91.94	87.42	92.50	86.62
	FIVES	93.50	91.49	90.36	91.06	89.72	87.26
ConvNeXt (43)	OIHC	95.54	93.29	92.26	91.47	93.48	88.02
	FIVES	93.39	91.25	90.08	90.98	89.53	89.03
MambaOut (13)	OIHC	94.61	92.51	92.29	91.36	92.86	90.65
	FIVES	93.64	91.83	90.54	91.22	90.79	90.25
Dense-XGB (23)	OIHC	95.21	92.56	85.25	91.72	93.29	86.23
	FIVES	93.98	92.19	82.83	91.75	89.65	88.37
Iwin-B (44)	OIHC	95.02	92.31	92.08	87.93	92.25	85.58
	FIVES	92.83	90.85	89.80	90.54	89.39	87.24
DSA-mamba (45)	OIHC	95.60	92.90	92.86	92.20	93.99	91.48
	FIVES	94.00	92.36	90.94	91.71	90.62	90.86
Proposed	OIHC	96.32	94.41	94.05	93.08	94.61	92.73
	FIVES	95.10	93.57	92.99	92.90	93.05	92.02

**Figure 8:**
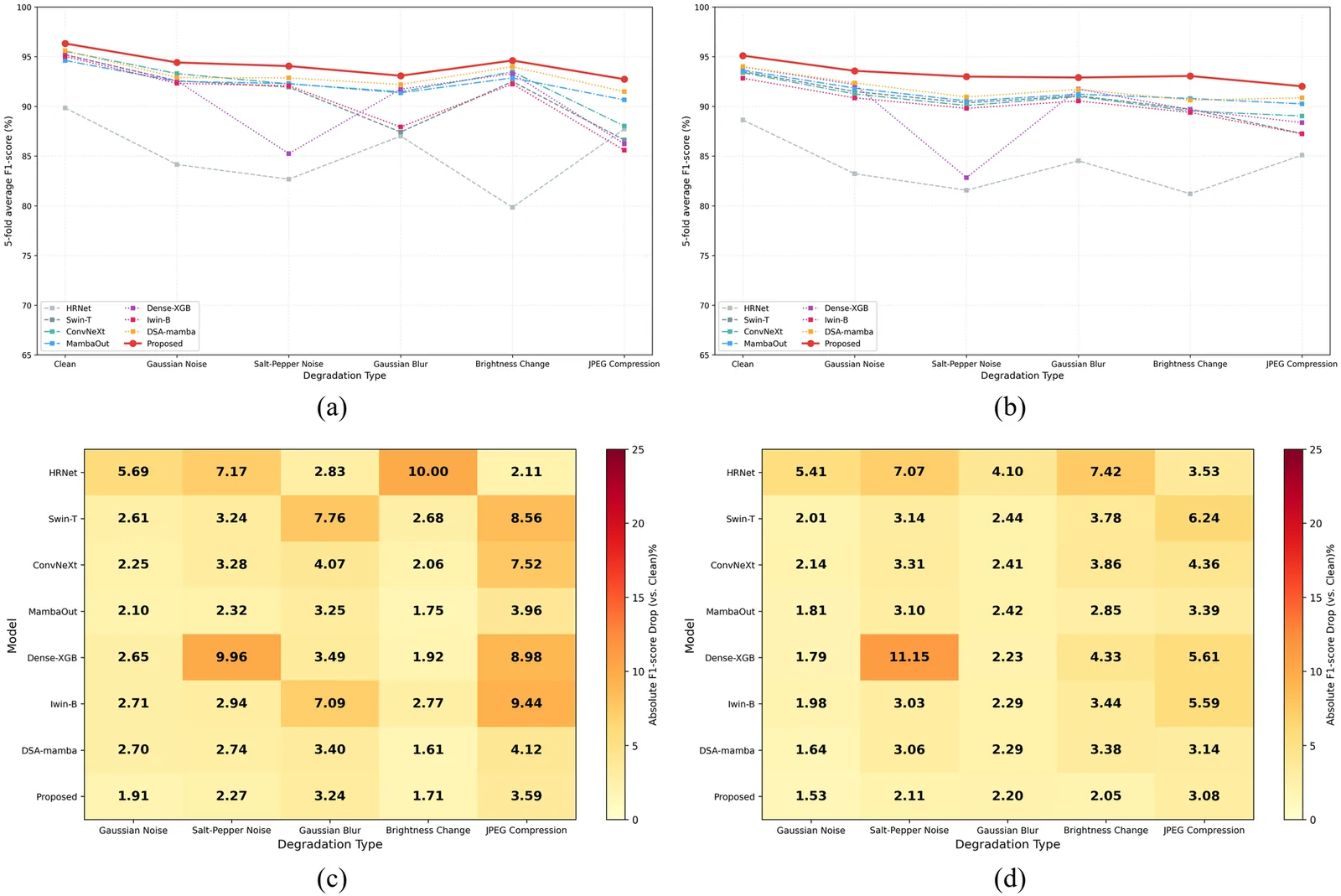
Performance trends and robustness analysis of different models under various image degradations on the OIHC and FIVES datasets. **(a,b)** depict the 5-fold average F1-score trends across different degradation types on the OIHC and FIVES datasets, respectively. **(c,d)** are heatmaps illustrating the absolute F1-score drop relative to the clean image. The colorbar on the right indicates the magnitude of the drop (darker colors represent larger drops).

Figures 8c, 8d visualize the F1-score drop relative to clean images for each model. Our model shows the smallest degradation across all five degradation types on both datasets, with drops ranging from 1.71% to 3.59% on OIHC and from 1.53% to 3.08% on FIVES. In contrast, several competing models exhibit substantially larger drops under specific degradations. On OIHC, Swin-T drops 7.76% under Gaussian blur and 8.56% under JPEG compression, while Dense-XGB drops 9.96% under salt-and-pepper noise. On FIVES, Dense-XGB drops 11.15% under salt-and-pepper noise, and HRNet drops 7.42% under brightness change. Robustness tests demonstrate that our model maintains relatively stable performance across both datasets under various types of image degradation.

### 4.6 Visualization analysis for the proposed model

Although deep learning models have shown promising performance in fundus disease classification, their black-box nature often makes the internal decision-making mechanisms difficult to interpret. Interpretability analysis using visualization techniques is often considered important for gaining clinician trust in model predictions. Thus, we applied Grad-CAM and LIME to visualize and interpret the decisions made by the model. The Grad-CAM technique generates heatmaps that highlight the critical image regions the model focuses on during classification. It allows verification of whether the model captures pathologically meaningful features, such as hemorrhages, exudates, or optic disc morphology. LIME is an explanation method based on local perturbation. It identifies which regions of an input image are most influential to the network's prediction. To generate explanations, LIME first partitions an image into superpixels or meaningful segments. It then produces multiple perturbed versions of the original input by randomly masking out certain segments. The masked regions are assigned neutral pixel values to observe how the model's prediction changes. In this way, LIME provides local explanations that help reveal which image features drive the model's decisions. Figure 9 presents Grad-CAM heatmaps, their overlays, and LIME visualizations across different disease categories. In Grad-CAM heatmaps, red and yellow regions indicate the highest attention, while blue and green represent background or non-critical areas with lower model attention.

**Figure 9:**
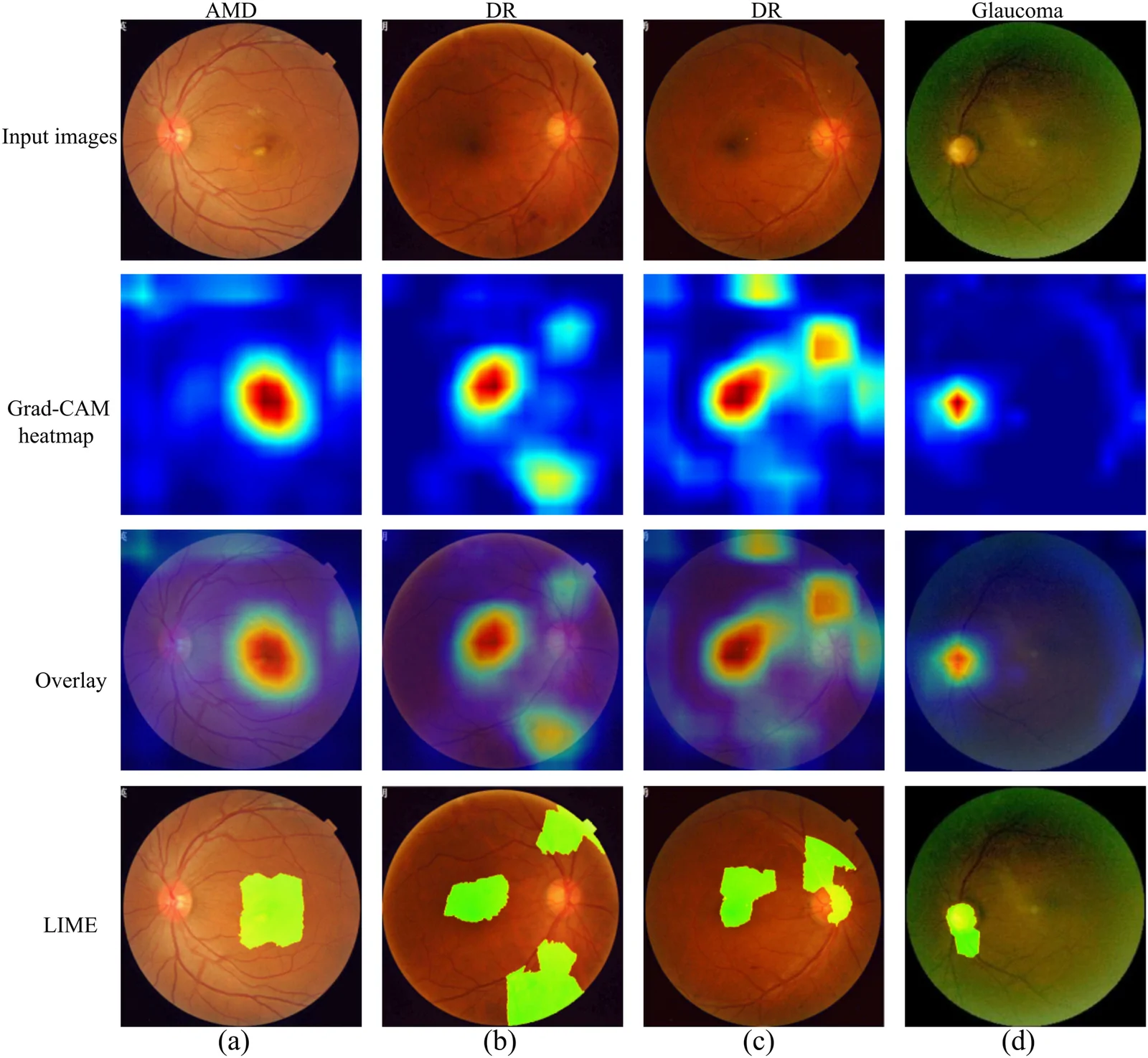
Visualization of model interpretability based on Grad-CAM and LIME. **(a)** Label: AMD, **(b)** and **(c)** Label: DR, and **(d)** Label: Glaucoma.

As depicted in Figure 9, the model's high-activation regions are located in anatomical structures relevant to disease pathology. In the AMD case shown in Figure 9a, the Grad-CAM heatmap covers lesions in the macular area, which is consistent with the pathological involvement of the macula in AMD. The LIME visualization further supports this observation by highlighting the same macular region. In Figures 9b, 9c, the heatmaps similarly focus on typical lesions such as hemorrhages and hard exudates. The LIME images for these DR cases exhibit green regions primarily in the macular area and inferior retina, which largely overlap with the Grad-CAM focal points. For glaucoma images, the model attends to the optic disc and cup regions, which are key anatomical structures used for clinical glaucoma assessment. The LIME result also highlights a focused green region tightly covering the optic disc and cup. The Grad-CAM and LIME results are mutually consistent and both correspond to clinically relevant features of glaucoma. This consistency supports the interpretability of the model's decisions and suggests that the model tends to focus on clinically meaningful pathological features.

## 5 Discussion and limitations

### 5.1 Overall performance and component contributions

The proposed framework achieved 97.03% accuracy and 95.89% Kappa on OIHC, and 95.13% accuracy and 93.49% Kappa on FIVES. These results suggest that combining contrastive pre-training with a hybrid dual-branch architecture holds promise for fundus disease classification. Several factors contribute to this performance. SimCLR-based pre-training learns discriminative representations from unlabeled data, reducing dependence on manual annotations. The dual-branch design combines EfficientNet-B4 for local texture capture and MambaOut for precise feature refinement with large-kernel convolutions. The ablation study shows steady improvement with each added module, indicating consistent learning with stable cross-validation performance. The t-SNE visualizations further show enhanced intra-class compactness and inter-class separability.

The ablation study reveals consistent gains from each component. The baseline EfficientNet-B4 achieved 95.39% accuracy on OIHC, and the progressive addition of the MambaOut branch, FDLC module, SimCLR pre-training, and dendritic classifier each contributed measurable improvements. With the FDLC module, the F1-score on OIHC rose from 95.01% (Model2) to 95.50% (Model4). The module adaptively weights the two branches for each sample and outperforms fixed weighting. SimCLR pre-training provided larger benefits when labels were scarce. With 25% labeled data, the gains reached 1.34 percentage points on OIHC and 1.58 percentage points on FIVES, suggesting that self-supervised learning mitigates annotation scarcity. The dendritic classifier further improved accuracy from 96.78% to 97.03% on OIHC and F1-score from 94.78% to 95.10% on FIVES. Its design enhances discriminative ability through non-linear decision boundaries, thereby contributing to the overall performance.

### 5.2 Robustness and interpretability

The proposed framework maintained stable performance under all five distortion conditions. On OIHC, F1-score drops ranged from 1.71% to 3.59%; on FIVES, from 1.53% to 3.08%. In contrast, Dense-XGB dropped 9.96% under salt-and-pepper noise on OIHC and 11.15% on FIVES, while HRNet fell 7.42% under brightness change on FIVES. These results indicate that the performance of some single-model approaches showed larger declines under certain challenging distortions. Meanwhile, our dual-branch framework maintains modest performance fluctuations under five distortion conditions. Such stability suggests that the combination of contrastive pre-training and dual-branch feature extraction offers a degree of resilience to common image quality degradations.

For interpretability, this study employs both Grad-CAM and LIME. Grad-CAM generates heatmaps highlighting critical regions. LIME provides local explanations through superpixel perturbation. The two techniques together offer a more complete picture of model behavior. The visualizations reveal consistent attention toward anatomically relevant structures. For AMD, Grad-CAM activation overlays the macular area, and LIME covers the same region. In DR cases, both methods highlight hemorrhages and exudates in the macular and inferior retinal areas. For glaucoma, Grad-CAM and LIME both center on the optic disc and cup, aligning with clinical assessment. The consistency between Grad-CAM and LIME visualizations suggests that the model attends to disease-relevant regional features.

### 5.3 Comparison with existing methods

The comparison experiments show that the proposed model exhibits competitive performance relative to existing approaches. On the OIHC dataset, the framework improved accuracy by 0.70 percentage points and Kappa by 0.98 percentage points over DSA-mamba. On the FIVES dataset, the gains over DSA-mamba reached 1.11 percentage points in accuracy and 1.49 percentage points in Kappa. In terms of computational efficiency, as shown in Table 8, the proposed model has 43.94M parameters, 6.06G FLOPs, and an inference time of 17.73 ms. Relative to lightweight models such as Swin-T and MambaOut, it requires more parameters and computation, as well as a longer inference time. Its efficiency is also slightly lower than that of DSA-mamba in terms of parameters and FLOPs, though inference time is comparable.

### 5.4 Limitations

Several limitations of this study should be acknowledged. First, in terms of computational efficiency, although the dual-branch architecture improves classification accuracy, its computational cost is relatively high compared with lightweight models such as Swin-T and MambaOut. This is mainly due to the parallel execution of two independent backbones, and the dendritic classifier also introduces additional parameter overhead. In resource-constrained scenarios, this efficiency issue may limit model deployment. Future work will explore strategies such as knowledge distillation, pruning, or more efficient branch designs to reduce computational cost. Second, regarding data processing, the OIHC dataset used in this work was constructed by integrating fundus images from multiple public sources. We systematically screened the dataset through perceptual hash comparison, patient information cross-validation, and manual review. However, some public datasets may share overlapping original sources. Given the limitations of the currently available metadata, we cannot completely rule out the possibility that a very small number of samples may have deeper-level associations. For the FIVES dataset and some subsets within OIHC, since the source datasets do not provide official patient mapping files, we performed inference-based grouping. This approach cannot guarantee strict patient-level isolation and may therefore lead to an overestimation of model performance. In addition, different classes in the OIHC dataset originate from different source datasets. These sources differ in imaging devices, camera types, and acquisition protocols, which may introduce domain bias and affect model learning. Moreover, some Normal cases share the same source as the ODIR-5K pretraining data, which may further affect the experimental results. Therefore, the results reported in this study should be interpreted under the limitations of the above data composition and splitting conditions. Meanwhile, due to constraints in data availability, our experiments were primarily conducted using internal 5-fold cross-validation, and the model has not yet been validated on an independent external dataset. As demonstrated by Duan and Tu (23), performance differences between internal and external validation can be substantial. Consequently, the generalizability of our model still requires further validation on multi-center and cross-device external datasets. Future studies should establish rigorous source-tracing mechanisms at the outset of dataset construction and adopt independently sourced pretraining and test datasets. This would allow for a more comprehensive assessment of true generalization capability. Third, regarding the ablation study, the performance improvements introduced by individual modules are relatively modest. Although paired t-test results indicate that these improvements are statistically meaningful, their practical clinical significance still requires further validation on larger-scale independent datasets. In addition, the current ablation study only compared the FDLC module with fixed-weight and dynamic-weight fusion strategies. A broader comparison with other fusion mechanisms, such as attention-based or confidence-based methods, has not been explored in this work and remains an important direction for future investigation. Fourth, the current classification framework is designed for mutually exclusive multi-class tasks, whereas in clinical practice, fundus diseases often involve co-existing pathologies, and the current model does not yet support multi-label classification. Extending the framework to multi-label scenarios represents a key direction for future work. Finally, although we employed Grad-CAM and LIME to provide visual interpretability of model decisions, the current analysis remains primarily qualitative. Future work incorporating quantitative assessment and clinician-involved validation would further enhance the clinical credibility of the model.

## 6 Conclusion

A unified framework integrating self-supervised contrastive pre-training with a hybrid dual-branch architecture is proposed to address multi-class fundus disease classification. This pre-training strategy enables the model to achieve competitive performance with limited labels, helping reduce dependence on fully annotated data. The dual-branch structure combines EfficientNet-B4 and MambaOut to capture semantic features. These features are adaptively fused by the FDLC module. A dendritic classifier is further introduced to enhance lesion discrimination ability. Experimental results on the OIHC and FIVES datasets demonstrate that the proposed method achieves the highest F1-score among the compared methods. Ablation studies indicate the contribution of each component to the overall performance. Grad-CAM and LIME visualization also show that the model's attention regions partially overlap with clinically relevant anatomical structures. These results provide preliminary evidence for the effectiveness of the proposed framework and motivate further refinement on broader datasets. Future work will extend the framework to true multi-label classification with co-occurring diseases. We will also validate the model on larger multi-centric datasets and explore model compression for resource-constrained scenarios.

## Data Availability

The original contributions presented in the study are included in the article/Supplementary Material, further inquiries can be directed to the corresponding author.
